# Burden for family carers at the end of life; a mixed-method study of the perspectives of family carers and GPs

**DOI:** 10.1186/1472-684X-13-16

**Published:** 2014-03-31

**Authors:** Maria C De Korte-Verhoef, H Roeline W Pasman, Bart PM Schweitzer, Anneke L Francke, Bregje D Onwuteaka-Philipsen, Luc Deliens

**Affiliations:** 1 Department of Public and Occupational Health & Expertise Center Palliative Care VUmc, EMGO Institute for Health and Care Research, VU University medical center (VUmc), P.O. Box 7057, 1007, MB Amsterdam, The Netherlands; 2 Department of General Practice, EMGO Institute for Health and Care Research, VU University medical center, Amsterdam, The Netherlands; 3 NIVEL, Netherlands Institute for Health Services Research, Utrecht, The Netherlands; 4 Ghent University & Vrije Universiteit Brussel, End-of-life Care Research Group, Brussels, Belgium

**Keywords:** Palliative care, General practitioner, Family carer, Burden, Hospitalisation, Mixed method

## Abstract

**Background:**

Since many patients spend most of the time at home at the end of life, this may affect the burden for family carers and constitute a risk factor for the patients’ hospitalisation. This study aimed to explore family carers’ burden in the final three months of the patient’s life, from the perspective of both carers and general practitioners (GPs), and to assess whether family burden, as defined by the GP, is associated with hospitalisation.

**Methods:**

A cross-sectional nationwide survey among GPs and family carers was performed. Participants were 194 GPs and 74 family carers of patients who died non-suddenly. Additionally, in-depth interviews were conducted with 18 family carers. For the quantitative analyses descriptive statistics, weighted Kappa and multivariate logistic regression analysis was performed. For the qualitative part thematic analysis was conducted.

**Results:**

The proportion of family carers experiencing a fairly heavy or severe burden increased significantly from 32% (second and third months before death) to 66% (one week before death). Most carers (95%) felt an emotional burden and 29% felt a physical burden in the final week. Three-quarters of carers did not perceive their burden as a problem because caring often felt rewarding. No significant association was found between the characteristics of family caregivers or professional care and the degree of family caregiver burden. Also, there was no significant evidence that patients of family carers for whom the GP assessed a fairly heavy to severe burden, were more likely to be hospitalised.

**Conclusions:**

The different overall assessment of family carers’ burden between GPs and family carers and the increasing emotional and physical burden of family carers towards the end constitute relevant information for GPs that will help them understand and anticipate carers’ personal needs.

## Background

Since many palliative care patients spend most of the time at home at the end of life [1,2], this may have an impact on the burden for family carers. Family carers may feel different kinds of burden at the end of the patient’s life: not just a physical burden, but also an emotional burden [3-7]. Burden has the potential to increase the carer’s vulnerability and may be a risk factor for burn-out [8].

In many countries, the General Practitioner (GP) is the key professional providing palliative care at home [9-12]. A guiding principle in palliative care is that the GP not only gives attention to the patient but also offers information and support to the family [13,14]. GPs usually pay frequent visits to the patient towards the end of life [15].

At the end of life, many patients experience a functional decline [16,17] which may have impact on burden for family carers. Therefore, it is important to explore how burden develops as the patient approaches death, and how family carers experience this. In addition, different studies found discrepancies in assessment of symptoms or quality in life from the physicians’ and family carers’ perspectives [18,19]. As a consequence of this, whether the GP assessment agrees with the family carers’ self-assessment is of interest in terms of family carers’ burden.

Several studies reported that living with relatives, extended family support, the ability of family carers to cope with the patients’ illness and low psychological distress among carers are positively associated with home deaths [20-22]. In a study of out-of-hours general practices, family carers’ burden was one of the reasons for hospital referral for 9% of palliative care patients who were referred to a hospital [23], but it is unknown whether carers’ burden is associated with hospitalisation at the end of life.

To explore family carers’ burden in the final three months of the patient’s life, from the perspective of both carers and GPs, to assess whether burden is associated with family carer characteristics, (professional) care and hospitalisation; the following research questions addressed in this paper are 1) What degree of burden and which types of burden do family carers experience during the final three months of a patient’s life and does the burden change as the patient approaches death? 2) Is the degree of family carers’ burden associated with characteristics of family caregivers and (professional) care? 3) What is the level of agreement between the self-assessment of burden by family carers and the assessment by GPs? 4) Is the degree of burden, defined and assessed by the GP, associated with hospitalisation in the final week of a patient’s life?

In this study we define a family carer as an unpaid person providing physical, practical and/or emotional care and/or support to a relative or friend.

## Methods

### Design

We conducted a mixed-method study, using a deductive sequential strategy [24]. Firstly, we conducted a cross-sectional nationwide quantitative survey among GPs and family carers in the Netherlands in 2011. Secondly, we held qualitative interviews among a selection of carers in order to explore the answers given in the quantitative survey in more depth.

### Study population

#### Quantitative study

The present study about family carers’ burden at the end of life is part of a larger study about hospitalisations at the end of life. For this larger study, a random sample of 2000 GPs was selected from 8896 registered GPs in the 2010 Dutch “Medical Address book”. In the questionnaire we asked the GPs to recall the last adult patient who died non-suddenly in the past year. Of the 2000 GPs in the sample, 238 were not working as a GP when they received the questionnaire and 161 had not had a patient who met the inclusion criteria. This resulted in 1601 eligible GPs. 222 GPs (14%) responded to the first mailing; the total response after follow-up mailings was 598 GPs (37%). We only asked the GPs about carers’ burden in the first mailing. Also, it was only in this first mailing that we asked the GPs to send a letter to the patient’s main family carer. In this letter, the carer was invited to participate in the research. In total, 121 GPs sent a letter to the carer. Of the 101 GPs who did not send a letter to the carer, 28 stated that they did not know the carer’s address and 31 considered the questionnaire too burdensome. Of the 121 carers approached, 83 completed the questionnaire. For the present paper we excluded 28 of the 222 GP questionnaires and 9 of the 83 carer questionnaires, since they reported on patients who had not resided at home most of the time in the last three months of life. This left 194 GP questionnaires and 74 carer questionnaires for the present paper.

#### Qualitative interview study

A total of 28 family carers indicated that they would be willing to participate in the interview study. Based on the questionnaires family carers had filled in, we were able to select purposively 18 of the 28 eligible carers. Purposive selection was based on diversity in degree and type of burden, age, patients’ disease and whether or not the patient was hospitalised.

### Measurement

#### Quantitative questionnaire study

The written questionnaires for GPs and family carers were developed using relevant literature [3-7] and open interviews with five doctors and three carers. A draft of the questionnaire was tested on face validity among 14 GPs and six carers. Their comments were incorporated in the final version of the questionnaires.

The family carers’ questionnaire consisted of several parts, one of which concerned the burden experienced. Although family carers’ burden is often seen as multi-dimensional [4,6,7], for this study we chose one general question. Family carers were asked “Overall, how burdened did you feel?” in three periods: in the second and third months before the patient’s death, in the second to fourth weeks before death and in the final week on a four-point scale (‘not/hardly at all’, ‘somewhat’, ‘fairly heavy’ and ‘severe’). After this general question, we asked about two types of burden, distinguishing between an emotional and a physical burden, and whether the burden was perceived as a problem to them. The GP questionnaire included the same general question about the degree of burden experienced by family carers. GPs were also asked if and when the patient was hospitalised. Hospitalisation was defined as staying in a hospital for at least one night.

#### Qualitative interview study

Of 18 open in-depth interviews, 15 face-to-face interviews were held at the family carer’s home and three were conducted by phone. The mean interview time was one hour. The open interviews started with a ‘grand tour’ question: “Tell me about the situation of your relative in the last six months of life”. Answers given in the questionnaire study were used to probe more deeply into this experience; for example, “You filled in that you didn’t experience the burden as a problem - can you say more about that?” These open interviews were conducted by an experienced interviewer (MDK) in the first half of 2012.

### Analyses

#### Quantitative questionnaire study

The degree and type of burden experienced by family carers (n = 74) were analysed using descriptive statistics with a confidence interval of 95%. A Chi-square test (categorical data) or independent T-test (continuous data) were used to assess the significance of differences between family carers who experienced a high level of burden and family carers who experienced a low level of burden with regard to caregiver and care characteristics. To assess the level of agreement between the GPs’ assessment of family carers’ burden and the self-assessment of the burden by the family carers, we checked the relationship of the family carer to the patient in the GP questionnaire with that in the corresponding family carer questionnaire. We concluded that nine did not match and they were therefore excluded in this analysis (giving n = 65). For these dyads a weighted Kappa was calculated.

A multivariate logistic regression analysis was performed to test the hypothesis that GPs’ assessment of family carers’ burden is associated with hospitalisation in the final week of life. For this analysis we used the data of all responding GPs and used the GPs’ assessment of the family carers’ burden in the second to fourth week before the patient’s death, so that we are sure the burden already existed before the hospitalisation.

#### Qualitative interview study

The verbatim transcribed interviews were analysed using qualitative data analysis [25]. The first transcripts were read thoroughly and the first codings were discussed by two researchers experienced in qualitative research (MDK and HP). Then a coding scheme was conceived based on the answers given in the questionnaire study and the new themes we found in the transcripts. Then the coding scheme and interview transcripts were entered in the software program Atlas-ti. The relevant interview fragments were linked to codes and we tried to find fragments that confirmed or contradicted the quantitative findings. This analysis process was conducted by MDK and step by step discussed with the co-researcher (RP).

#### Ethics

A study protocol was approved by the Ethics Board of the VU University Medical Center Amsterdam. Before the start of each interview, the interviewee was told that participation was voluntary, that the transcripts would be anonymous and that confidentiality was assured. After that, an informed consent form was signed by the family carer.

## Results

### Family carer and GP characteristics

The mean age of family carers (n = 74) was 62, with a range from 40 to 86. About two thirds of them were female and 41% were employed. The relationship to the patient was that of partner (70%), daughter/son (19%), sibling (4%) or other (7%). The mean age of the GPs (n = 194) was 50, with a range from 31 to 64; 57% were male and 68% had been trained in palliative care. In the qualitative part of the study the mean age of family caregivers (n = 18) was 59, with a range from 44 to 82. Furthermore, 83% were female and the relationship to the patient was that of partner for 72% of them and daughter/son for 22%. Of the18 interviewed family carers six (33%) experienced a fairly heavy to severe burden in the second and third months before death and twelve (66%) one week before death.

### Degree of burden

The percentage of family carers who experienced a fairly heavy to severe burden increased significantly from 32% (in the second and third months before death) (CI: 22%-45%) to 66% (one week before death) (CI: 54%-77%) (not in tables). The increasing proportion of carers who felt a fairly heavy or severe burden as the patient came closer to death was often explained in the interviews as due to an accumulation of symptoms and physical deterioration as the patient’s death approached; therefore the patient needed more care (Table 1, quote 1).

**Table 1:** Interview fragments of family carers’ burden and hospitalisation (Family carers’ perspective in interviews, n = 18)

	**Quotations**
	**I = interviewer**
	**F = Family carer**
**Degree of burden**	**Quote 1** F15 (Woman 47 caring for partner, felt fairly heavy burden in the three time periods)
	F: Well, at first he managed all right - he could make a coffee himself, I mean, and he could take a shower on his own and all that kind of thing. But there comes a point when you see that he needs more and more help and you just feel “I don’t know how much longer this is going to go on for but I just want to be there for him for those last few months we’ve got”.
	**Quote 2** F09 (Woman 48 caring for partner, felt no burden in the three time periods):
	I: Didn’t you find it hard going?
	F: No. I slept on the sofa for a year so well, you just do that, you just do that. (…) But this, well, I don’t know, I looked after him and made sure he got what he wanted right up to the end and so, then there’s a feeling of satisfaction that I did it properly, that’s what you wanted and that makes it all right.
**Type of burden**	**Quote 3** F30 (Woman 44 caring for partner, felt severe burden in the three time periods)
	I: You said that it was an emotional burden in the final months?
	F: Yes, all the time, yes.
	I: Can you say more about that? What was the reason?
	F: Well, I think it’s only logical, you see, you know you’re going to lose your husband. But I have to say I was in a daze the whole time, what with the hospital visits, the kids. It seems as if you kind of put your thoughts - your feelings - to one side a bit so that you’re there for him and don’t make things any worse.
	**Quote 4** F29 (Woman 62 caring for mother, felt some burden in the 2^nd^ and 3^th^ month of patients’ life and fairly heavy burden in the last month of patients’ life.)
	I: Some people feel an emotional burden at a certain point because they think things are really not going well. Did you have that problem at all?
	F: No, I always felt that we should be pleased she had lived as long as she did because she’d always been quite poorly and had things wrong with her heart and so on. So we used to say things like we hadn’t expected her to make 90 or 91 with all the problems she’d always had and so on. So it wasn’t that I got very emotional about it, I think I’m too down-to-earth for that anyway, you know. We have to be grateful she lived as long as she did.
	**(Quote 5)** F23 (Man 61 caring for partner, felt severe burden in the three time periods):
	F: Well, someone who's fully involved in life and doing all kind of things is obviously going to feel incredibly cut off if they lose that ability little by little. (…). She was bothered about those things. And you can’t do anything about it, you can’t… You see, if someone’s hungry you can give them something to eat. If someone’s thirsty you can give them something to drink and if someone’s sad you can put an arm around them. But you can’t do anything about that. You can’t give someone back what they’ve lost.
**Problem of burden**	**Quote 6** F29 (Woman 62 caring for mother), felt some burden in the 2^nd^ and 3^th^ month and life and fairly heavy burden in the last month of patients’ life.)
	F: They [parents] did a lot for me in the past, so that I could carry on working, and now I’d like to repay that. But of course that doesn’t stop it sometimes being a burden.
	**Quote 7** F23 (Man 61 caring for partner: felt severe burden in the three time periods):
	F: Well, I think it’s the confirmation for her, you know, that you are prepared to keep going 24 hours a day for her because you love her, you really love her. But it’s also confirmation for you personally: look, I love that woman so much that this is what I’m prepared to do. And then it’s not about making the effort, that’s not even quite what it is - perhaps it’s difficult to explain. I think it’s significant, it has added value if you have to make an effort.
	I: For who?
	F: Well, for the relationship I think. I do see it that way
**Burden and hospitalisation**	**Quote 8** F25 (Woman 61 caring for a friend, felt no or hardly no burden in the 2^nd^ and 3^th^ month, some burden in the 2^nd^ to 4^th^ week, and fairly heavy burden in the final week of patients’ life )
	F: Yes, the GP thought - saw - that it was getting too much for me as well. Because when he was admitted to (NAME of peripheral hospital), she put so much effort into finding him a place somewhere so that I could get my breath back again.
	I: Did that help you, getting your breath back?
	F: No, it didn’t. Because not only did I have to go to the hospital every day, that man was also waiting for me there every day. Yes, just like a child waiting for his mum. Kind of “Mummy, I’ve missed you”. Because that man didn’t have anyone else left (cries), I was the only one giving him love and caring for him.
	**Quote 9** F09 (Woman 48 caring for partner, felt no burden in the three time periods):
	F: And then one or two weeks in between in hospital and then he’d phone me at 7 in the morning saying can you come and wash me because it takes so long, and they always hurt me, and then I’d spend the entire day in the hospital.
	**Quote 10** F04 (Woman 63 caring for partner, felt severe burden in the three time periods):
	I: What did the nurses do?
	F: The nurses washed and shaved my husband and had a chat with me over coffee.
	I: Did the nurses give you support?
	F: Yes, they did give me support because they told how everything was going.
	I: Did you also consider having night-time care?
	F: Yes, I tried it for two nights. Because I normally slept on the sofa in the room where he was sleeping. Just when I dropped off to sleep he would get up quietly to go to the toilet but he wasn’t able to stand properly. When we had night-time care I slept in my own bed, but I kept waking up. No, it didn’t help; I wanted to be with my husband because I didn’t know when he would die. I wanted to be with him when he died. It was hard going. But I’d always promised him I would look after him at home

The overall degree of burden many family carers mentioned could on the one hand be perceived as severe while on the other hand they found it rewarding to be doing something for their loved one; giving good care was the last contribution they could make (Table 1, quote 2). Because of this rewarding feeling, some carers did not describe the burden as fairly heavy or severe in the questionnaire even though they said that they were putting a great deal of effort into caring for their relative.

### Type of burden

Of the family carers who felt at least some burden, most experienced emotional burden during the three time periods (85%, 92% and 95%, see Table 2). In the interviews, many carers said that knowing the relative was going to die made it an emotionally burdensome period. They felt the loss that was approaching when they looked at their loved one (Table 1, quote 3). Some of the carers did not feel a severe emotional burden because their relative had become very old with deteriorating health; in the carer’s opinion their time had come to pass away (Table 1, quote 4). Also, many family carers mentioned that they felt an emotional burden from watching the patient’s suffering. This was not only due to the accumulation of symptoms in the patient, but also due to feeling powerless when seeing the social, mental and physical identity fade away (Table 1, quote 5).

**Table 2:** Degree and type of burden as experienced by family carers during the final three months of life (family carers, n = 74*)

	**2**^**nd**^**and 3**^**rd**^**months before death**	**2**^**nd**^**to 4**^**th**^**weeks before death**	**Final week**
**Degree of family carer burden**	% (CI 95%)	% (CI 95%)	% (CI 95%)
Not/hardly at all	23 (14–34)	10 (4–19)	8 (3–17)
Somewhat	45 (33–57)	36 ( 25–48)	26 (16–37)
Fairly heavy	23 (14–34)	33 (22–45)	38 (27–50)
Severe	10 (4–19)	22 (13–33)	28 (19–40)
**Type of burden**			
**(only for family carers with some to severe burden)**			
Emotional	85 (72–93)	92 (82–97)	95 (87–99)
Physical	25 (14–38)	27 (17–40)	29 (18–41)

*Between 0–3 missing observations per period.

A significantly smaller proportion of family carers experienced a physical burden in the three time periods (25%, 27% and 29%, see Table 2). Care was mostly felt to be a physical burden when the patient was bedbound and needed care in the form of bathing, dressing and toileting. Carers also mentioned the burden of interrupted sleep, communicating with professionals and family, monitoring the patient and being available 24 hours a day.

### Problem of burden

Across the three time periods, burden was not perceived to be a problem for 75% of family carers, although one third felt a fairly heavy to severe burden in the second and third months before death and two thirds of them felt a fairly heavy to severe burden in the last month before death (not in Table). In the interviews, many family carers said that it was normal to care for and support their partner or parent because previously the loved one had cared a lot for them, and therefore the burden did not feel like a problem (Table 1, quotes 6 and 7). This principle of reciprocity was a view held by many family carers but not all of them. The burden was perceived to be a problem when the caring and interrupted sleep continued for a long time without there being a clear idea when it would end.

### Association between the characteristics of family caregivers or professional care and the degree of family caregiver burden

For family carers’ age, gender, relation to the patient, employment of family carers, number of family caregivers, number of GP visits and received nursing care no significant association was found between family carers with a high level of burden and a low level of burden during the second to fourth weeks before the patient’s death (Table 3). For the other two time periods, one week before death and second and third months before death we found no significant associations (not in table).

**Table 3:** **Association between the characteristics of family caregivers or (professional) care and the degree of family caregiver burden in the 2**^**nd**^**to 4**^**th **^week before death (n = 74)*

	**Fairly heavy to severe burden (n = 40)**	**No to some burden (n = 31)**	
	**%**	**%**	**P-value**
**Family caregiver characteristics**			
Main family carer			
Age (mean, SD)	63 (SD 13)	62 (SD 11)	0.50
Gender, male	27	32	0.66
Partner of patient	70	68	0.83
Employed	43	43	0.94
Number of other caregivers			0.18
No other family carers	11	30	
1-2 other family carers	53	33	
3-5 other family carers	21	23	
> 5 other family carers	16	13	
**Characteristics of care**			
GP visits			0.60
No visits	5	4	
1 visit	34	46	
2 or more visits	61	50	
Nursing care	85	69	0.12

*Three missing observations.

### GPs’ assessment of family carers’ burden

**Figure 1:**
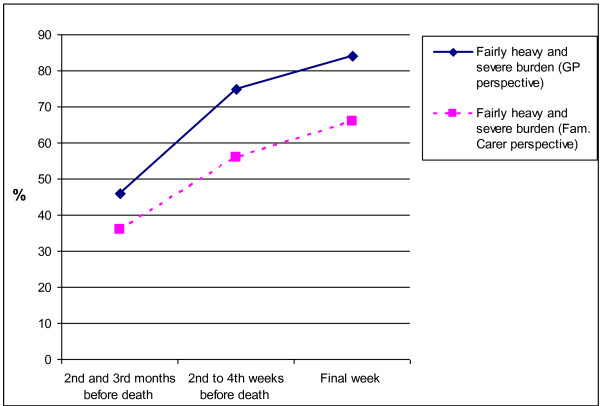
Proportion of family carers and GPs who assessed family carers’ burden to be fairly heavy or severe in the final three months of the patients’ life (Dyads of family carer and GP perspectives, n = 65).

GPs were more likely to assess family carers as having a fairly heavy or severe burden than the family carers’ self-assessment (Figure 1). In the three periods, the assessment of the burden by GPs agreed with that by family carers in 32%, 35% and 30% of cases respectively (Table 4). A large proportion of GPs estimated family carers’ burden to be higher during the three periods (35%, 41% and 47%); the opposite of this, a lower estimation of family carers’ burden, was found for 30%, 24% and 24% of the dyads respectively. The level of agreement was poor at two to three months before death (Kappa = 0.19) and poor at two to four weeks before death (Kappa = 0.13). No weighted Kappa coefficient could be calculated for the final week because observed concordance was smaller than mean-chance concordance.

**Table 4:** Assessment of family carer burden and level of agreement (Dyads of family carer and GP perspectives, n = 65*)

		**Family carer perspective**				
**Second and third months before death**		**Not/hardly at all**	**Somewhat**	**Fairly heavy**	**Severe**	**Total**
**GP**	Not/hardly at all	3 (4.8%)	7 (11,3%)	1 (1.6%)	0	11 (17.7%)
**perspective**	Somewhat	6 (9.7%)	9 (14.5%)	7 (11.3%)	0	22 (35.5%)
	Fairly heavy	2 (3.2%)	11 (17.7%)	7 (11.3%)	5 (8.1%)	25 (40.3%)
	Severe	0	2 (3.2%)	1 (1.6%)	1 (1.6%)	4 (6.5%)
		11 (17.7%)	29 (46.8%)	16 (25.8%)	6 (9.7%)	62 (100%)
**Second to fourth week before death**		Kappa with linear weighting 0.19 (CI 95% 0.02-0.35)				
**GP**	Not/hardly at all	0	0	1 (1.6%)	0	1 (1.6)
**perspective**	Somewhat	1 (1.6%)	6 (9.5%)	5 (7.9%)	2 (3.2%)	14 (22.2%)
	Fairly heavy	4 (6.3%)	14 (22.2%)	9 (14.3%)	7 (11.1%)	34 (54%)
	Severe	0	3 (4.8%)	4 (6.3%)	7 (11.1%)	14 (22.5%)
		5 (7.9%)	23 (36.5%)	19 (30.2%)	16 (25.4%)	63 (100%)
		Kappa with linear weighting 0.13 (CI 95% 0–0.29)				
**Final week**						
**GP**	Not/hardly at all	0	0	2 (3.1%)	0	2 (3.1%)
**perspective**	Somewhat	3 (4.7%)	1 (1.6%)	3 (4.7%)	1 (1.6%)	8 (12.5%)
	Fairly heavy	1 (1.6%)	9 (14.1%)	7 (10.9%)	9 (14.1%)	26 (40.6%)
	Severe	1 (1.6%)	7 (10.9%)	9 (14.1%)	11 (17.2%)	28 (43.8%)
		5 (7.8%)	17 (26.6%)	21 (32.8%)	21 (32.8%)	64 (100%)
		A linear Kappa cannot be calculated because observed concordance is smaller than mean-chance concordance				

*Between 1–3 missing observations per period.

### Association between GPs’ assessment of family carers’ burden and hospitalisation in final week of life

In a multivariate analysis of patients who spent most of the time at home, after correcting for age and living alone, no significant association was found between the GPs’ assessment of the degree of family carers’ burden during the second to fourth weeks before the patient’s death, and patient hospitalisation in the final week (Table 5).

**Table 5:** Association between GPs’ assessment of family carers’ burden during second to fourth week before patients’ death and hospitalisation in the final week (GP perspective, n = 194)

	**Total (n = 194)**	**No hospitalisation in final week (n = 161)**	**Hospitalisation in final week (n = 33)**	
	%	%	%	Odds (CI 95%)
No to some burden	31	30	36	1
Fairly heavy burden	46	45	49	0.76 (0.32-1.78)
Severe burden	23	24	15	0.42 (0.13-1.34)

*Multivariate analysis after correction for age and living alone.

In the interviews, some family carers said that the burden was one of the reasons for hospitalisation. Although the physical burden was reduced for many family carers due to the patient receiving 24-hour professional care in the hospital, sometimes they continued to care for the patient. In addition, hospitalisation could also give rise to other burdens, for example feeling responsible for checking the professional care such as the provision of medication or dealing with inattentive professionals (Table 1, quotes 8, 9). The shift from one type of burden to another type was also mentioned when more care was arranged at home. Other family carers, community nurses and night-time care could often relieve the family carers’ physical burden. But sometimes this help was perceived more as an extra emotional burden than as relief (Table 1, quote 10).

## Discussion

### Statement of principal findings

In this study we looked at the unique combination of family carers’ and GPs’ perspectives on family carers’ degree, type and change of burden in the last three months of the patient’s life and whether this is associated with hospitalisation. The proportion of family carers experiencing a fairly heavy to severe burden increased from one third during the second and third months before death to two thirds in the final week. Most carers felt an emotional burden and a smaller proportion experienced a physical burden. Three-quarters of carers did not perceive their burden as a problem. The interviews showed that this was explained by the fact that caring for and supporting their relative often felt rewarding and it was the final thing they could do for their loved one. No significant association was found between the characteristics of family caregivers or professional care and the degree of family caregiver burden. Also there was no significant evidence that patients of family carers of whom the GP assessed a fairly heavy to severe burden, were more likely to be hospitalised.

### Strengths and weaknesses

A strength of this study is the new insights provided in how the burden for family carers changes as patients come closer to death, from the perspective of both carers and GPs, and the association with hospitalisation. The generalisability of the findings may be limited because of the low response rate of GPs and carers. The reason for the low GP response rate may be the complex procedure of asking the GPs to invite carers to participate in the research. However there is little difference between the mean age and gender breakdown in our GP sample and the general population of Dutch GPs, for whom the mean age is 48 and where 59% are male [26]. Half of the GPs did not invite the carers to participate in the study; 31% of these GPs did not do so because they thought it would be too burdensome for the carers. Because of this, there may be an underestimation of the family carer burden. A further limitation is the retrospective design of the study. In assessing the burden of family carers, recall bias cannot be excluded from both the family carer him/herself as from the GP. However, because both GPs and carers filled in the questionnaire retrospectively, we don’t know in which direction this might have affected the outcome. Our results should be interpreted with caution because no validated questionnaire is used. However, our one item question about degree of burden is comparable with the one item overall question about burden in the ‘Zarit Burden Interview’ which was tested as a useful screening instrument [7].

### Family carers’ burden and support

Many family carers perceived the burden not to be a problem. Many carers said in the interviews that caring felt rewarding and important to do because it was the final thing they could do for their loved one. These positive feelings of reward and the perceived value of the care they provide help carers to cope with the situation were confirmed in in-depth studies with a cross sectional design [4,8,27]. The importance of coping with the situation may explain why many carers did not feel their burden to be heavy even when they spent a great deal of effort on caring for their relative in objective terms. In addition to this, our research shows that although a proportion of 66% family carers experienced a fairly heavy to severe burden in the last week of life, only a proportion of 25% perceive burden as a problem. Therefore it is recommended to GPs not only asking about the degree of burden, but also to ask whether the burden is perceived as a problem for the family caregiver.

### GPs’ assessment of family carers’ burden

An important result of this study is that GPs tended to assess the burden to be higher than family carers did in their self-assessment. In more than one third of the cases, the GPs estimated the burden to be higher, although in more than one fifth of the cases they estimated the burden to be lower. Given the above-mentioned results that carers do not always perceive their burden to be as high as one would expect on the basis of care activities, it is debatable whether these higher GP estimations are an overestimate rather than the lower family carer assessment being an underestimate. Overestimation by GPs might be a problem if GPs’ assessment of family burden is a reason for hospitalisation. However, in this study no evidence was found that the likelihood of hospitalisation was higher for patients for whom the GP had assessed fairly heavy or severe family caregiver burden. However, in some interviews it was explained that hospitalisation was chosen as a solution to give respite to the carers, especially for those who had cared for a long time. But then other kinds of burden could arise.

## Conclusions

In order to understand the overall degree and development of burden and needs of family carers, it is important for GPs to discuss the multi-dimensionality of burden and the felt problem of burden regularly with carers, so that they can anticipate family carers’ personal needs. More research is needed to know when, and for whom, the burden becomes excessive for family carers at the end of life.

## Competing interest

The authors declare that they have no competing interest in preparing this study.

## Authors’ contributions

All authors read, revised and approved the final manuscript. HP and LD initiated the study and obtained the funding. MDK conducted the interviews, analysed the interviews and provided the draft of this paper. The first transcripts and the first codings were discussed by MDK and HP. MDK, HP and AF discussed the analyses of the qualitative interviews and MDK, HP and BOP performed the statistical analyses. HP, BOP, BS, AF and LD supervised the project.

## Pre-publication history

The pre-publication history for this paper can be accessed here:

http://www.biomedcentral.com/1472-684X/13/16/prepub
